# A qualitative study of patient education needs for hip and knee replacement

**DOI:** 10.1186/s12891-017-1769-9

**Published:** 2017-10-12

**Authors:** Deborah Kennedy, Amy Wainwright, Lucy Pereira, Susan Robarts, Patricia Dickson, Jennifer Christian, Fiona Webster

**Affiliations:** 1 Sunnybrook Holland Orthopaedic and Arthritic Centre, 43 Wellesley Street East, Toronto, ON M4Y 1H1 Canada; 2 School of Rehabilitation Science, McMaster University, 1400 Main St. W, Hamilton, ON L8S 1C7 Canada; 3 Department of Physical Therapy, University of Toronto, 500 University Avenue, Toronto, ON M5G 1V7 Canada; 4 Department of Occupational Science and Occupational Therapy, University of Toronto, 500 University Avenue, Toronto, ON M5G 1V7 Canada; 5 Centre for Addiction and Mental Health, 33 Russell Street, Toronto, ON M5S 2S1 Canada; 6 Institute of Health Policy Management and Evaluation, Dalla Lana School of Public Health, University of Toronto, 155 College Street, Toronto, ON M5T 3M7 Canada

**Keywords:** Patient engagement, Hip and knee replacement, Education, Pain management, Person-centred care

## Abstract

**Background:**

Quality health information is key to patient engagement, self-management and an enhanced healthcare experience. There is strong evidence to support involving patients and their families in the development and evaluation of health-related educational material. These factors were the impetus for our high volume joint replacement centre to undertake a qualitative study to elicit patient experiences to inform the development of effective strategies and education along the care continuum for hip and knee replacement.

**Methods:**

Purposively selected patients from postoperative follow-up clinics were recruited to participate in a focus group or telephone interview. We developed a semi-structured interview guide that addressed four specific aspects of the patient’s experience with educational material: pre-surgery, hospital stay, recovery period and future recommendations. The focus groups and interviews continued to the point of saturation and were audio-recorded and transcribed verbatim. Interview transcripts were coded and then inductively organized into larger categories using thematic analysis.

**Results:**

Six focus groups and seven telephone interviews were conducted, totalling 32 participants. One of the key themes that emerged was a need for more education concerning pain management post-operatively; specifically, patients wanted more information on expected levels of pain, pain medication usage, management of side effects and guidelines for weaning off the medication. There was surprising variability in patients’ descriptions of their pre-surgery, surgery and recovery experiences. These corresponded to an equally diverse range of preferences for educational content, delivery and timing. Many patients reported using the web while others preferred traditional formats for information delivery. There was some interest in receiving education using mobile technology.

**Conclusions:**

Our findings validate the importance of multi-modal patient education tailored to individual preferences and experiences, which may differ according to such characteristics as gender and age. The gap in pain management information is a critical finding for healthcare providers working with patients undergoing joint replacement. Developing pain management education in different formats that addresses frequently asked questions will enhance patient engagement and, their overall experience and recovery.

**Electronic supplementary material:**

The online version of this article (10.1186/s12891-017-1769-9) contains supplementary material, which is available to authorized users.

## Background

In our increasingly complex healthcare environment, patients require high quality information to successfully manage their health. Although access to information on the web and other sources has increased, it has not necessarily translated to increased understanding. Often patient health information has been created from the lens of the providers without patient consultation.

In 2015, the World Health Organization released a global strategy on people-centred and integrated health services calling for a fundamental shift in the way health services are managed and delivered [1]. The current focus for healthcare organizations is a person-centred care approach, where the patient’s values, knowledge, preferences and needs are central to their care. There is strong evidence to support the importance of involving patients and their families in the development and evaluation of educational material for health purposes [2, 3].

Currently, there is limited literature exploring the education needs of patients and their families in many patient populations including that of hip and knee replacement. This information would be extremely useful in the design of effective strategies that support and educate patients both prior to, in hospital and after discharge. Given the growing prevalence of this surgery, along with shortening lengths of stay, patients need this type of information for shared decision-making.

These factors were the impetus for our team to undertake a qualitative study at our high volume joint replacement centre and seek the voice of our patients about their educational needs and preferences. Our study question was, “What are the informational needs and delivery preferences for education of families and patients undergoing hip or knee replacement?”

## Methods

### Design and setting

The study was conducted in a large Canadian orthopaedic centre specialized in joint replacement surgery. Using descriptive qualitative methods [4], purposefully selected patients were recruited from outpatient clinics at their 6 week to one-year follow-up visits post joint replacement.

### Sampling

Using purposeful sampling, a small percentage of patients were invited to participate in the study to explore their experiences and preferences for education following hip or knee replacement surgery [5]. Maximum variation sampling was used to ensure we had participants who differed by age, gender, affected joint, and marital status. This helped to ensure that the patients we interviewed were similar to those regularly seen at our facility. We did not collect information about socio-economic status. We conducted focus groups as well as telephone interviews to accommodate those who did not reside within the urban setting of the hospital. One spouse attended a focus group at the participant’s request as she did not feel sufficiently comfortable with English but wanted to share her story. One focus group was composed entirely of men. Six focus groups and seven telephone interviews were conducted totalling 32 participants, at which point our team decided we had generated sufficient data to reach saturation while maintaining a manageable final dataset within this project’s timeframe. The mean age of the 32 participants was 67.9 (standard deviation ±7.82; min 46, max 78) years. There was equal distribution of men and women (*n* = 16 each) and similarly, hip and knee replacements. Regarding time point of participation, 44% of the patients were up to 3 months postoperatively, another 44% between 3 months to 9 months and 12% greater than 9 months up to 1 year.

### Ethics

Local Research Ethics Board approval was obtained for this study. Patients were approached for consent during their follow-up visits. The clinic secretary asked the patient if they would be willing to receive a consent form, and have someone from research speak to them about participating in a focus group or interview.

### Focus groups

We developed a focus group guide to address four specific aspects of the patient’s experience with educational material [6]. The guide (see Additional file 1) began with open, broader questions about the patient’s educational needs and experiences leading up to surgery and then questions were asked about each stage of the hospital and recovery process. Finally, a series of questions were asked in relation to the patient’s preferences for future educational materials, including videos and internet resources. All questions were meant to be exploratory and relied on probes to allow differences between patients in perceptions and experiences to emerge during the course of the interview. The focus group guide was pilot tested with one patient identified by our research team. The objective of pilot testing was to confirm the length of each interview and to ensure that the questions were clear and comprehensible. FW, who is a trained qualitative interviewer, performed the pilot interview with one patient and facilitated several of the focus groups and telephone interviews; other focus groups were conducted by an experienced Research Associate, JC. Members of the healthcare team did not solicit or conduct interviews. All interviews and focus groups were audio-taped and professionally transcribed verbatim.

### Data analysis

Data collection and analysis were conducted concurrently. Three members of the research team (DK, FW and JC) read transcriptions of the pilot interview independently to identify codes. The researchers then met to compare their independent analyses and develop a coding framework for use in the subsequent analysis. JC then coded the remaining interviews, keeping memo notes of any potential changes to the coding framework that she identified. After all interviews had been coded, the larger research team held several meetings in which they interpreted the data to identify similarities and differences across the interviews. We combined our codes into themes and identified predominant ones. The relationships between the themes were also summarized in these team meetings.

Our team performed several steps to ensure rigour [6]. To ensure *reliability*, more than one investigator performed each key step. Our analysis was *reflexive* as our team was multidisciplinary, consisting of clinicians, educators and researchers, which allowed us to consider our personal biases during data analysis throughout the memoing and data summarizing steps [6].

## Results

Although some people preferred to be interviewed one-on-one rather than in a focus group, we did not find any significant differences in their accounts. For the purposes of this study, using both data collection approaches allowed us to achieve both depth and breadth, increasing the number of participants we could speak with over a relatively short period of time [7]. While qualitative interviews and focus groups are not inter-changeable we did not find significant differences in the data we obtained across using the two approaches. We organized our findings into four main themes: 1) education gaps relating to pain management 2) participant’s validation of existing organizational education materials; 3) informal sources of information; and 4) interest in new delivery modes for education, such as mobile health applications.

### Theme 1: Educational gaps around pain management

An important finding that emerged from our interviews and focus groups was an identified need to have more education around pain management post-operatively. In particular, participants expressed an interest in education related to expected levels of post-operative pain, the purpose of the prescribed medications, information on how to take the medications, their side effects and how to “wean off” pain medications. In the following account, the patient describes how she needed more information to manage her pain,

*“Well, if there was possibility to deal with the pain part more effectively … that might have been of help. I think [the surgeon] did say if I had any questions … I could phone them, I just don’t think I did because I thought there wasn’t really much they could do about, you know, the pain stuff …”* (Participant #1, Interview #4, Female)

Another participant describes his perception of other people’s negative experiences of medication side effects. In the following excerpt, he is suggesting that patients are both afraid to ask about medication and are without recourse regarding pain medication once they leave the hospital. He says,

*“I mean, I got off those white little pills as fast as I possibly could. I took Tylenol Extra Strength for whatever, and now maybe once a week or so I’ll take a couple Tylenol Extra Strength. But two things, one is a lot of the people that go to rehab, they get sick from them, they get sick from those white little pills, whatever they are, OxyContin or oxy-something, they get sick, but the doctor told them to take them and they continue to take them … but there’s no recourse back, and they’re afraid to ask, they’re definitely afraid to ask about the pills … So again it’s education about medication.”* (Participant #1, Focus Group #1, Male)

Participants suggested that nobody provided them with information about how to “wean” themselves off their pain medication once they were back at home.

*“…The first time around I expected someone to say to me, “Once you get your pain medication here’s a method of getting off of it. Here’s a way of weaning yourself off of that,” but nobody did this. …, it was maybe at six weeks I was still taking Oxy. The first time [the pharmacist] go, “You gotta stop doing this,” I go, “What do you mean?” No one had told me [that]. … even this time when I had my hip nobody would give me a method of weaning myself off of it, I had to go back to the pain specialist.”* (Participant #3, Focus Group #1, Male)

While participants did acknowledge they received information that they would need to reduce their pain medication, they frequently felt that these instructions lacked crucial information about how they would accomplish this in terms of practical steps. As one participant shared,

*“I think I started to wean off on my own without realizing it. It was every four hours, I think, if I remember. And then I realized afterwards I had forgotten one time, and I thought, “Oh, okay, I’ve been an hour without it,” so I took it then because I felt pain. So the next day I really tested it out … Now, I was fortunate … But it was a nurse here, before I left, when I got the prescription, who said to me, “You’ll want to wean yourself off,” but not how.”* (Participant #6, Focus Group #1, Female)

### Theme 2: Participants’ validation of existing materials

Despite identifying a gap in pain management education, patients did validate the usefulness of the existing patient education tools (www.sunnybrook.ca/holland) offered in different mediums both pre and post-operatively. These included a comprehensive guide (organized into preoperative, hospital stay and post discharge sections), a preoperative education class, as well as, a short animated educational YouTube video developed by our organization in partnership with Dr. Mike Evans. Of note, not all participants attended the workshop or recalled seeing the video.

#### 2a) guide for patients having hip or knee replacement

The following participant highlights that the Guide is useful across various stages of the pre to post surgery process and was something she referred to throughout her recovery.

*“I like having the book because you don’t remember everything you read the first time, and you don’t remember everything a month later, so I could go back to the book, especially about preparing and getting ready. So it was nice to have the printed material that I could look at.”* (Participant #1, Interview #2, Female)

#### 2b) the preoperative education class

Participants commented on the benefits of attending the Preoperative Education class. They commented on how appropriate preparation *“built their confidence”* (Participant #1, Focus Group #1, Male). This confidence building was important given how many people were initially fearful of having surgery. In the following account, the participant stresses the importance of hearing from another patient and described how valuable it was to have the expertise of the rehabilitation staff.

*“I was terrified like I think most people are, and there was a person who’d gone through the surgery who was able to answer any questions that most people had. The physiotherapist and the OT were, you know, incredibly gracious and able to sit down and really, you know, relax most of the patients there.”* (Participant #1, Pilot Interview, Female)

#### 2c) Dr. Mike Evans’ ‘preparing you for your hip or knee replacement surgery’ (www.sunnybrook.ca/holland/hipknee)

Participants who had viewed the Dr. Mike Evans’ video expressed that they had found it helpful, especially as it emphasized and was consistent with the information they had received in the Guide.

*“…I think it repeated a lot of what was in the pamphlet, but you could see it. You know, it’s concrete, tangible, you see it, very step-by-step, and so it helped me…”* (Participant #2, Focus Group #4, Female)

### Theme 3: Favoured sources of patient information

Patients identified several sources of information that they drew on most frequently. Not surprisingly, and consistent with other studies, these included family and friends; information they found on the internet (referred to by several participants as “Dr. Google”) as well as surgeons.

#### 3a) family and friends

Patients frequently identified friends and families as an important source of information. Hearing stories from other people that their surgeries had been successful seemed to go a long way toward reassuring participants, as the following account exemplifies,

*“I think my bigger source was other people that had the same operation…what you do is you gather information, which is all part of the decision-making process, but you gather information from multiple sources and you start to get confident in the things that are consistently heard from everybody, so that’s where I got my information.”* (Participant #1, Focus Group #2, Female)

At the same time, they voiced their concern that experiential accounts were not necessarily medically valid. Related to this, many voiced a desire to access a bank of patient “testimonials” that the hospital could curate, hence increasing its reliability from a patient perspective. Several noted that they wanted to hear both “good and indifferent” experiences from others:

*“What I think is going to be helpful for me is testimonials, good and indifferent … so testimonials from past patients who have had whatever type of surgery, whether it’s hip or knee …”* (Participant #3, Focus group #2, Male)

#### 3b) “Dr. Google”

The majority of participants had searched Google for information on the surgery or recovery process. Many did not question the validity or accuracy of this information. For some participants, mostly men, they wanted to actually watch the surgery “*to see what goes on in the operating room*”.

Women were more likely to indicate that they specifically did not want to view the surgery online, as the following quote exemplifies:

*“I just kind of Googled “total knee replacement surgery” or, “required devices required after total knee replacements.” “… I didn’t look up what the procedure looked like because I thought I might chicken out then.” “No, I don’t want to see that!””* (Participant#1, Interview #4, Female)

Not all people thought that accessing information on the internet was useful and in some instances it reinforced the fear they already felt about the upcoming surgery.

#### 3c) surgeons as a source of education

Some participants identified the surgeon as their main source of information. While patients felt that surgeons were an important source of knowledgeable information, they often described mixed experiences of how much time they felt surgeons could or did provide.

### Theme 4: Interest in new delivery modes for education, such as social media

Several participants were interested in accessing information from newer technologies including mobile health applications and social media. A small number of people said that they would in the future use an app. As one participant noted,

*“[It would be] easy access to information to compare notes with other patients who’ve gone through it … either from apps or …Twitter* …” (Participant #4, Focus group #2, Male)

Other participants, however, were uncertain as to how social media would be useful for them. Some noted that they were comfortable with the computer but did not own smartphones or other technology that would enable them to use newer forms of social media/mobile apps. The following quotes were typical of what we heard during the focus groups.

*“I use a computer extensively and I look up a lot of stuff on the Internet, so a website that I can go to is fine, I just don’t do the social media, I don’t want to get involved, and I don’t have a smartphone, don’t want one. So for me, you know, that’s just my level and my choice. But it might benefit somebody else. We’re not dealing too often with younger people who are more into those things.”* (Participant #3, Focus Group #3, Female)*“I’m not sure. I’m still learning all these apps. Right now it’s information overload.”* (Participant #6, Focus Group #1, Female)

## Discussion

Our experiences and findings with this study have validated the importance of engaging our patients to fully understand their educational needs and delivery preferences. The largest gap that patients identified related to pain medication education, especially for the time-period after they had left the hospital and were at home recovering. This finding was very interesting to the team as historically, we had been very intentional to include information about pain management in our comprehensive guide and preoperative education classes. However, upon reviewing our materials, we realized that although we covered some core concepts, we did not provide enough details around specific medications or weaning steps.

Chan et al. [8] found that more than half of patients after knee replacement reported the first 2 weeks at home as being the most painful time-period after surgery. Poorly managed pain decreases patient satisfaction, the ability to progress functionally [8, 9] and has been found to increase the likelihood of persistent pain following joint replacement [10]. Chan et al’s study of over one hundred patients following knee replacement, after hospital discharge found there was suboptimal use of pain medication and non-pharmacological strategies, misconceptions of pain medication usage, disturbing side effects and inadequate information provided to manage pain at home [8]. Similarly, our patients voiced a need for more information on pain medication usage, side effects and how to wean off at the appropriate time. Given the shortening lengths of stay, these topics are becoming even more important as patients are managing acute pain while at home.

The importance of multiple modalities (pamphlets, classes, videos, Apps) available to patients in plain, clear language has been demonstrated in other populations [11]. Our findings also supported the importance of providing multi-modal education to patients that can be tailored to their individual learning preferences and experiences. Prior research investigating the information needs of patients undergoing arthroplasty found that although a core set of questions could be defined; the information needs across patients was quite variable [12, 13]. While this idea is not novel, we still have to broaden our understanding of the social and cultural differences between patients and how to address these in education.

Participants valued the organization’s educational materials, including the comprehensive guide, the YouTube video and the pre-operative education classes. The guide enabled patients to review materials at different stages of recovery. All participants described using this guide repeatedly along the continuum of care. The pre-operative education session was highly regarded and reduced fears by enabling patients to ask questions, clarify issues and interact with others undergoing similar surgeries. Several studies have shown the importance of preoperative education in reducing anxiety and improving satisfaction, however study findings are variable as demonstrated in a recent meta-analysis [14–16].

Other sources of information patients accessed included; families and friends, the internet and their surgeons. They strongly favoured learning from the experiences of other patients; often these were friends. For this reason, many suggested that having access to a curated set of patient vignettes or “stories” available on a hospital website would be useful. This would allow them to learn from peer experiences while having the added benefit of providing legitimacy to these accounts, an issue many struggled with in relation to the medical validity of the lay accounts they heard from others or by browsing the web. There was interest in using technology to deliver education such as mobile health apps. Although, not all patients have access to smartphones or tablets, the use of mobile technology is increasing annually. The latest Government of Canada statistics indicated that in 2014, 66% of Canadians owned a smartphone and 49% owned a tablet [17].

The study findings have been the impetus for a number of quality improvement projects. We have strengthened the role of patient and family advisors to improve the care experience. To address the gap in our pain management education, patient advisors and team members co-designed new materials in multimodal formats. We introduced a new brochure and video addressing the top 10 questions about pain medication after joint replacement, the information of which is also available online (www.sunnybrook.ca/hipkneepain). Concurrently, we provided widespread staff education about the new resources.

These experiences have reinforced the importance of including the patient voice, as the healthcare team’s focus was not always reflective of patients’ preferences. We have also launched a mobile app (with a web-based version to follow shortly) that includes a daily health check with recommendations based on responses and a library of resources on key patient identified topics to empower patients to better manage their recovery. The new pain management information is located in the education library of the mobile app.

Our study had several limitations. Due to resource implications, we recruited only English speaking patients (except in one instance in which an English-speaking spouse participated); future studies might focus on non-English speaking patients. We did not specifically target younger patients for whom the use of mobile technology and social media might be more relevant. Finally, we did not collect data about individual participant’s demographic details such as home support, occupation, marital status and level of education and therefore did not stratify our analysis across these differences. We did however obtain rich, in-depth information about the experiences of our patients, with the goal of providing insight into their experiences, rather than on obtaining statistical generalizability. For this reason, the core concepts from our research should be transferable to other centres wanting to develop more patient-centred education materials.

## Conclusion

Patients undergoing hip and knee replacement have a wide range of preferences for educational content, modes of delivery and timing to successfully engage and manage their recovery. The findings of our study underscore the importance of multi-modal patient education that can be tailored to individual preferences and experiences. It is important to offer traditional formats for information delivery as well as alternatives using web and mobile technology.

Patients emphasized the need for more comprehensive education concerning pain management following discharge from hospital. Management of acute postoperative pain remains a significant challenge, particularly for those undergoing knee replacements. Given the trend of shortening lengths of stay, patients are coping with acute pain at home; therefore, they require more information on pain medication usage, side effects and guidelines for weaning off the medications. Since completing this study, our site has introduced a new pain management brochure and video, which are also available online and through our new mobile App.

## Additional file

Additional file 1: Semi-structured Interview Guide. (DOCX 14 kb)
