# Supplementary material for: A qualitative study of patient education needs for hip and knee replacement
Source: BMC Musculoskelet Disord. 2017 Oct 12;18:413. doi: 10.1186/s12891-017-1769-9 (PMC5639777; doi:10.1186/s12891-017-1769-9)
Supplement: Additional file 1: — Semi-structured Interview Guide. (DOCX 14 kb) [file 12891_2017_1769_MOESM1_ESM.docx]

**Supplementary File**

**Semi-structured Interview Guide**

We are interviewing you to better understand your experiences as a patient or family member who has undergone surgery for hip or knee replacement surgery and as a consequence received educational materials. Are there any questions about what I have just explained?

_________________________________________________________________________

**General description of experience with health informational needs**

Why don’t we start by going around the group and having everyone say a few words about why you decided to join the focus group today and a little about when your surgery was? What were your information needs?

**Sources of educational materials needs prior to surgery**

Thinking back to your decision to have surgery, can you talk about where you received information about the surgery? Prompts: friends, family members, health care providers (specific), internet searches, etc. How did you assess the quality of these sources of information?

Was there any information you couldn’t find or weren’t provided that would have been helpful at this stage?

**Educational materials received at the Holland Centre**

Now I’d like to talk a little bit about the resources you were given at the Holland Centre. Can you describe what they were (Guide booklet, video). Which of these resources did you find the most useful? Why/why not? Which of these resources did you find the least useful? Why/why not?

**Use of educational materials post-surgery**

Once you were home, did you continue to find the resources helpful? What times of information did you most need during this time period?

**Future resources**

Thinking about the various technologies available now, are there any that you’ve used to get health information? Any you’ve heard of that you’re interested in but haven’t used?

If available would you be interested in using any of the following:

- Desktop and mobile Apps
- social media sites (i.e. blogs, Twitter, facebook)
- telemedicine

**Recommendations**

We would like to explore with you what advice you would give to other patients. What source or type of information was most valuable to you?

Prompts: Can you please describe why?

**Conclusion**

- Is there anything else that you would like to comment on?
